# Neurological Manifestations and Diagnostic Delay in Pediatric Isolated Sphenoid Sinus Disease: A Systematic Review

**DOI:** 10.3390/brainsci16080897

**Published:** 2026-08-21

**Authors:** Eryk Latoch, Natalia Sufin, Weronika Samełko, Michał Płoński

**Affiliations:** Department of Neurology and Pediatrics, Medical University of Bialystok, ul. Waszyngtona 17, 15-274 Białystok, Poland; 41486@student.umb.edu.pl (N.S.); 41481@student.umb.edu.pl (W.S.); michal.plonski@udsk.pl (M.P.)

**Keywords:** isolated sphenoid sinus disease, neurological manifestations, neuro-ophthalmological symptoms, cranial nerve palsy, diagnostic delay, pediatric neurology, secondary headache, sphenoid sinusitis

## Abstract

**Highlights:**

**What are the main findings?**

- Pediatric isolated sphenoid sinus disease (ISSD) frequently presents with neurological or neuro-ophthalmological manifestations, while sinonasal symptoms are often absent or subtle.
- Headache was the most common manifestation, but ocular symptoms, cranial nerve involvement, and frequent initial misdiagnosis indicate a broader neurological diagnostic phenotype.

**What are the implications of the main findings?**

- Isolated sphenoid sinus disease should be considered in children with persistent or atypical neurological symptoms, particularly when headache is accompanied by ocular symptoms or cranial nerve deficits.
- Early cross-sectional imaging with CT and/or MRI may reduce diagnostic delay and help prevent potentially serious neurological and orbital complications.

**Abstract:**

**Background**: Isolated sphenoid sinus disease (ISSD) in children is an uncommon but clinically significant condition that may present primarily with neurological or neuro-ophthalmological symptoms rather than typical sinonasal complaints. Owing to the anatomical proximity of the sphenoid sinus to the optic nerves, cavernous sinus, and multiple cranial nerves, even localized sphenoid pathology may produce symptoms that mimic primary neurological, neuro-ophthalmological, or headache disorders, contributing to diagnostic delay. **Objective:** To characterize the spectrum and frequency of neurological and neuro-ophthalmological manifestations, diagnostic pathways, treatment strategies, and reported clinical outcomes in pediatric ISSD, with particular emphasis on diagnostic delay and persistent neurological sequelae. **Methods:** A systematic review was conducted in accordance with the PRISMA 2020 guidelines. PubMed and Web of Science were searched from database inception to 3 January 2026. Observational studies reporting pediatric patients aged ≤18 years with radiologically confirmed ISSD were included. Single-patient case reports, reviews, and studies without extractable pediatric data were excluded. Owing to substantial clinical and methodological heterogeneity, findings were synthesized descriptively. Risk of bias was assessed using Joanna Briggs Institute (JBI) critical appraisal tools. **Results**: Eleven studies comprising 136 pediatric patients were included. Most cases were inflammatory lesions (*n* = 121, 89%), followed by mucoceles (*n* = 10, 7.3%) and tumors (*n* = 5, 3.7%). Headache was the most frequent neurological manifestation reported in 129 patients (94.9%). Ocular manifestations were present in 33/136 patients (24.2%), whereas otolaryngological symptoms were reported in 25/134 patients (18.7%). Cranial nerve involvement was documented in 10 out of 54 patients with available data (19%), most commonly affecting cranial nerves III, V, VI, and VII. Among 79 reported initial diagnoses, primary headache disorders were the most common (49.4%), whereas sphenoid sinusitis was correctly identified in only 20.3%. Diagnosis was established using CT and/or MRI in all included cases. Antibiotic therapy was the most common treatment for inflammatory lesions, while all patients with mucoceles and tumors underwent surgical management. Full recovery was reported in 81% of inflammatory cases and in all patients with mucoceles, although follow-up reporting was heterogeneous. **Conclusions**: Pediatric ISSD frequently presents with neurological and neuro-ophthalmological manifestations, whereas sinonasal symptoms may be absent or subtle. Persistent or atypical neurological symptoms, particularly headache accompanied by ocular symptoms or cranial nerve deficits, should prompt consideration of sphenoid sinus pathology and early cross-sectional imaging.

## 1. Introduction

Neurological symptoms in children often require careful differentiation between primary neurological disorders and secondary causes related to extracranial pathology. Isolated sphenoid sinus disease (ISSD) in the pediatric population is an uncommon and clinically significant condition that remains diagnostically challenging due to its rarity and often non-specific presentation. In contrast to other forms of rhinosinusitis, nasal symptoms are frequently absent or subtle, which may contribute to delayed recognition [1].

The sphenoid sinus is centrally located within the skull base and lies in close proximity to critical neurovascular structures, including the optic nerves, cavernous sinus, pituitary gland, and cranial nerves III, IV, V1, V2, and VI [2,3]. Consequently, even a localized pathological process may result in serious complications, particularly when diagnosis is delayed. Reported sequelae include meningitis, visual impairment, vascular complications, and persistent cranial nerve deficits [1,4,5,6].

In children, anatomical and developmental factors may further influence disease presentation. The sphenoid sinus undergoes gradual pneumatization and reaches full development later than other paranasal sinuses, which may affect both symptomatology and disease patterns [7]. Despite this, pediatric-specific data on ISSD remain limited, with most evidence derived from small case series or mixed populations.

According to the International Classification of Headache Disorders (ICHD-3), headache attributed to disorders of the nose or paranasal sinuses represents a recognized category of secondary headache [8]. Nevertheless, ISSD remains an underrecognized etiology of secondary headache in children, particularly when sinonasal symptoms are absent [9]. More broadly, ISSD may present with neurological or neuro-ophthalmological symptoms that initially obscure its sinonasal origin.

Advances in imaging have improved detection, with computed tomography (CT) serving as the primary modality for assessing bony anatomy, while magnetic resonance imaging (MRI) is essential for evaluating intracranial and orbital involvement, particularly in patients with neurological symptoms [10,11].

Neurological manifestations may represent the initial or predominant presentation of ISSD in children and are associated with a risk of significant morbidity. These patients are often first evaluated by neurologists rather than otolaryngologists, and the diagnostic process frequently begins in pediatric neurology settings. Although non-neurological symptoms may be present, neurological features often determine the diagnostic pathway and may contribute to delays in diagnosis [1].

The predominance of neurological or ocular symptoms may additionally contribute to misclassification as primary neurological, neuro-ophthalmological, or headache disorders.

The primary aim of this systematic review was to characterize the spectrum and frequency of neurological manifestations in pediatric ISSD, as well as to evaluate diagnostic pathways, treatment strategies, and long-term outcomes, with particular emphasis on persistent neurological sequelae.

## 2. Materials and Methods

### 2.1. Search Strategy

We conducted a systematic review in accordance with the PRISMA 2020 guidelines [12]. The review protocol was not prospectively registered in PROSPERO. The review was designed to investigate a rare and clinically heterogeneous pediatric condition for which standardized diagnostic criteria for ISSD are lacking. We acknowledge that prospective registration would have further strengthened methodological transparency; therefore, detailed eligibility criteria and the full reproducible search strategy are provided to facilitate assessment and replication of the review methodology.

**Figure 1:**
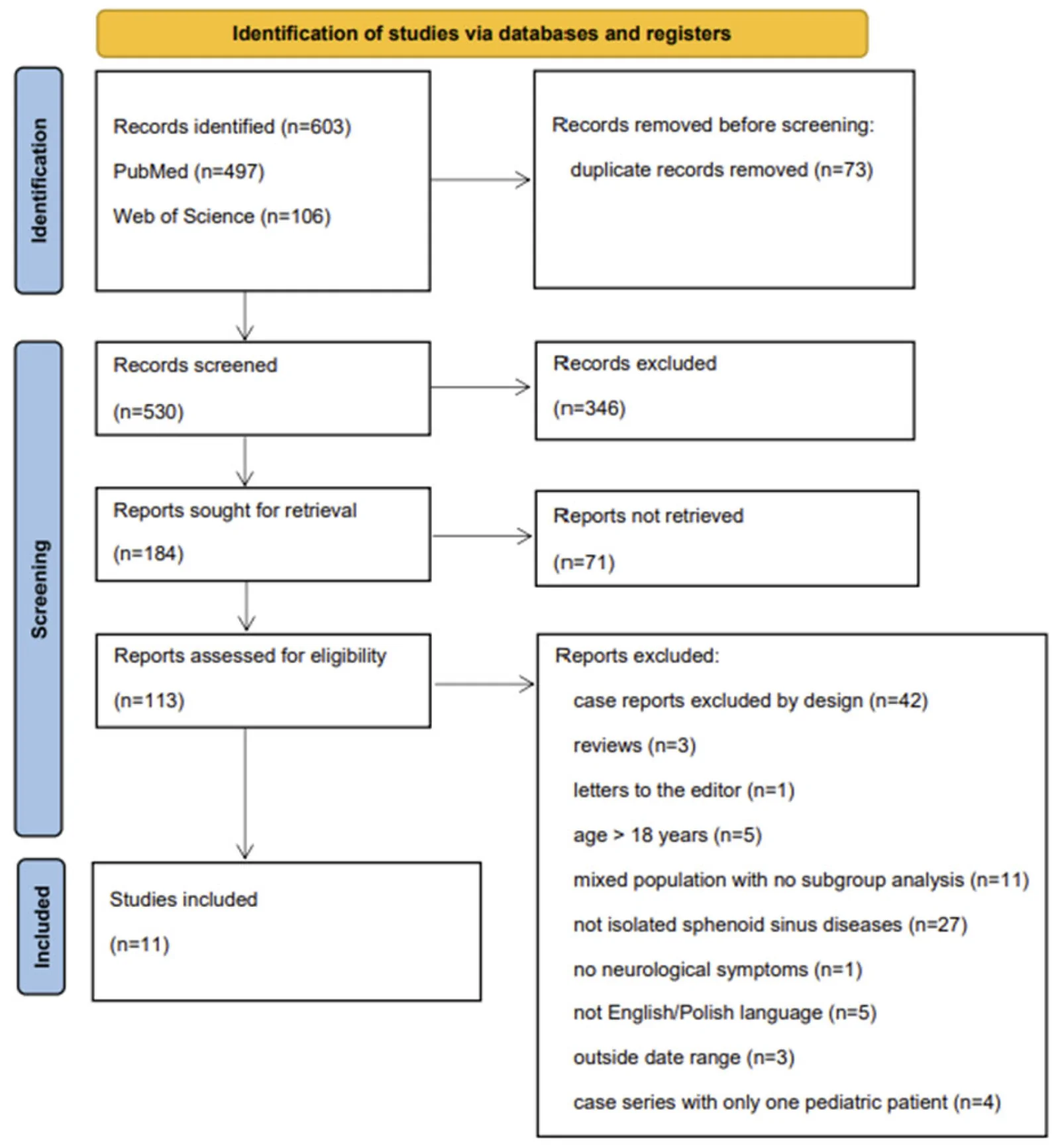
PRISMA 2020 flow diagram for new systematic reviews which included searches of databases and registers only.

We searched PubMed and Web of Science from database inception to 3 January 2026. No initial language restriction was applied; however, only studies published in English or Polish were included in the final analysis. The search strategy combined MeSH terms and free-text keywords using Boolean operators (AND/OR). The full reproducible search strategy for each database is provided in Supplementary Data S1. The study selection process was documented using a PRISMA 2020 flow diagram (Figure 1).

### 2.2. Inclusion Criteria

To ensure a structured and transparent selection process, eligibility criteria were established in accordance with the PICO framework. The study population included pediatric patients aged 18 years or younger with radiologically confirmed (CT and/or MRI) isolated sphenoid sinus disease. These criteria were designed to ensure that the analysis was based on symptomatic cases in which headache and neurological manifestations were directly attributable to isolated sphenoid sinus disease.

Due to the absence of a standardized definition of isolated sphenoid sinus disease (ISSD) in pediatric patients, a pragmatic approach was applied. EPOS 2020 provides general criteria for pediatric rhinosinusitis but does not specifically define isolated sphenoid sinus inflammation [11]. Therefore, the diagnosis of sphenoid sinus inflammation was accepted according to the criteria used by the original authors of each included study, provided that radiological imaging (CT and/or MRI) confirmed involvement of the sphenoid sinus. Where sufficient imaging data were available, studies were assessed to verify the absence of significant involvement of other paranasal sinuses.

The primary outcomes were the spectrum and frequency of neurological manifestations, including headache, ocular symptoms, cranial nerve involvement, intracranial/orbital complications, diagnostic delay, and long-term neurological sequelae. Secondary outcomes included treatment strategies and clinical outcomes. Particular emphasis was placed on headache characteristics, ocular manifestations, cranial neuropathies, and factors contributing to diagnostic delay or misclassification as primary headache disorders.

To ensure radiological diagnostic accuracy, eligibility was restricted to studies published from 1990 onward. Before 1990, the limited accessibility of CT and MRI meant that sphenoid pathology was often underdiagnosed or misdiagnosed using conventional sinus X-rays. Restricting the publication date aligns directly with our inclusion criterion of radiologically confirmed disease. Eligible study designs included observational studies, such as case series, cross-sectional studies, and case–control studies.

### 2.3. Exclusion Criteria

Duplicate publications reporting overlapping data, review articles, letters to the editor, and single-patient case reports were excluded. The exclusion of single-patient case reports was intended to reduce clinical and methodological heterogeneity and avoid the overrepresentation of rare, atypical, or severe neurological manifestations.

Studies published in languages other than English and Polish were not considered for final analysis. In accordance with the PICOS framework, studies including mixed adult and pediatric populations were excluded unless pediatric-specific data could be clearly extracted.

Articles lacking sufficient clinical detail, particularly regarding presenting symptoms, were excluded. Studies were excluded if presenting symptoms were attributable to paranasal sinuses other than the sphenoid sinus, or if neurological manifestations occurred in the context of systemic, metabolic, hematological, developmental, or primary osseous disorders in which sphenoid sinus involvement was considered secondary, incidental, or due to mass effect rather than primary sinonasal pathology. This encompassed sellar and parasellar developmental lesions (e.g., Rathke’s cleft cysts), primary skull base bone disorders such as fibrous dysplasia of the sphenoid bone, and structural anomalies including pneumosinus dilatans.

Studies were further excluded if sphenoid sinus pathology resulted from congenital malformations, recent craniofacial trauma, or prior iatrogenic interventions. Finally, patients with a history of malignancy were included only if sphenoid sinus disease was clearly independent of contiguous tumor spread, a systemic neoplastic process (such as acute lymphoblastic leukemia), or treatment-related effects.

### 2.4. Data Analysis

Records were imported into Zotero (version 7.0.30) and duplicates were removed. A standardized data extraction form was used to ensure consistency across reviewers. Title and abstract screening, as well as full-text assessment, were performed independently by W.S. and N.S. Discrepancies were resolved by consensus, with the involvement of a third reviewer (E.L.) when necessary.

Data were extracted on study characteristics, pediatric population demographics, initial suspected diagnoses, risk factors, etiology, clinical presentation, time to diagnosis, complications, management, and long-term outcomes. To avoid double counting, headache, ocular manifestations, focal cranial nerve deficits, and other neurological symptoms were analyzed as separate categories whenever possible. Since ISSD is an anatomical diagnosis rather than an etiological one, inflammatory, cystic, and neoplastic lesions were analyzed separately, to the extent that the available data permitted.

For variables with missing data, frequencies were calculated using the number of patients with available data as the denominator. For initial diagnosis, because more than one diagnosis could be reported for an individual patient, the denominator was the total number of reported initial diagnoses.

No formal statistical analysis was performed; findings were synthesized descriptively.

### 2.5. Quality Assessment

Risk of bias was assessed using the Joanna Briggs Institute (JBI) Critical Appraisal Tools [13,14], with the appropriate checklist selected according to the study design. As the number of items varies across JBI tools, the proportion of positive responses was reported for descriptive purposes. No studies were excluded based on quality assessment; results were interpreted in the context of the identified risk of bias. Overall, the methodological quality of the included studies was limited, as most were retrospective case series with small sample sizes, heterogeneous reporting, and incomplete follow-up.

Due to substantial clinical and methodological heterogeneity among the included studies, meta-analysis was not considered appropriate. Instead, a structured narrative synthesis was conducted, focusing on clinical presentation, diagnostic pathways, complications, and treatment outcomes (Table S2).

## 3. Results

### 3.1. Study Selection

Based on our search criteria, we identified a total of 603 records from two databases: PubMed (*n* = 497) and Web of Science (*n* = 106). After the removal of 73 duplicates, title and abstract screening was performed. Subsequently, 184 records were sought for full-text retrieval, of which 113 full-text articles were available and assessed for eligibility. Finally, 11 studies met the inclusion criteria. The PRISMA flow diagram illustrates the study selection process (Figure 1).

### 3.2. Study Characteristics

Eleven studies comprising 136 pediatric patients were included in the analysis. Of these, 10 were case series and one was a case–control study. Study characteristics are summarized in Table S1a,b. Studies including both pediatric and adult populations were eligible if data specific to patients aged ≤ 18 years could be clearly extracted; studies in which pediatric data could not be disaggregated were excluded. Consequently, seven studies included exclusively pediatric patients, whereas four included mixed adult and pediatric populations.

Among all included patients, 67 (53.6%) were male and 58 (46.4%) were female. In the study by Caimmi et al., only the “severe” subgroup was included, as the non-severe group lacked sufficient diagnostic data required for this review. As a result, sex distribution was not reported for 11 patients; therefore, sex proportions were calculated based on the remaining 125 patients. The age of participants across the included studies ranged from 5 to 18 years.

### 3.3. Clinical Manifestations

Table 1 summarizes the extracted data for the primary variables of interest, with particular emphasis on headache and neurological manifestations. Symptoms were categorized as neurological (including specific cranial nerve involvement) and non-neurological manifestations. Additional variables included risk factors, initial diagnosis, time from symptom onset to diagnosis, identified pathogens or histopathological findings in tumor cases, treatment strategies, and imaging modalities. Long-term neurological sequelae were analyzed as the primary outcome.

**Table 1:** Comprehensive Summary of Symptoms, Diagnostic Evaluation, and Therapeutic Outcomes in the Included Literature.

Study	Cases	Initial Diagnosis	Time to Diagnosis	Headache	Focal Neurological Deficits	Neurological Symptoms (Other)	Ocular Symptoms	Intracranial/Orbital Complications	Non-Neurological Symptoms	CT/MRI	Microbiological / Histopathological Findings	Treatment	Predisposing or Associated Factors	Long-Term Sequelae
*Inflammatory (acute/chronic)*														
*Lahat* et al., *1997* [15]	*n* = 10	CNS infection (*n* = 4, 40%) migraine headache (*n* = 2, 20%) CNS tumor (*n* = 1, 10%)	1–8 days	headache (*n* = 10, 100%)	CN V (*n* = 2, 20%) CN VI (*n* = 1, 10%)	nuchal rigidity (*n* = 3, 30%) periorbital pain (*n* = 3, 30%) stupor (*n* = 2, 20%) mental status changes (*n* = 2, 20%)	unspecified ocular symptoms (*n* = 1, 10%)	none	fever (*n* = 10, 100%) nasal symptoms (*n* = 8, 80%)	CT	*S.aureus*	antibiotics (*n* = 10, 100%) surgery (*n* = 2, 20%)	swimming and diving (*n* = 3, 30%)	full recovery (*n* = 10, 100%)
*Haimi-Cohen* et al., *1999* [16]	*n* = 8	NR	5–29 days	headache (*n* = 8, 100%)	none	none	pseudopapilloedema (*n* = 1, 12.5%)	none	nausea (*n* = 1, 12.5%)	CT	NR	antibiotics (*n* = 8, 100%)	allergic rhinitis (*n* = 3, 37.5%) sinusitis prior to admission (*n* = 1, 12.5%) URTI (*n* = 1, 12.5%)	full recovery (*n* = 8, 100%)
*Uren* et al., *2003* [17]	*n* = 8	NR	3 days–6 weeks	headache (*n* = 8, 100%)	NR	nuchal rigidity (*n* = 2, 25%)	blurred vision (*n* = 4, 50%) diplopia (*n* = 2, 25%) photophobia (*n* = 2, 25%) visual field defect (*n* = 1, 12.5%)	meningitis and frontal lobe abscess (*n* = 1, 12.5%)	fever (*n* = 6, 75%) nasal symptoms (*n* = 4, 50%)	CT	S.aureus	analgesics/observation (*n* = 1, 12.5%) antibiotics (*n* = 7, 87.5%) surgery (*n* = 3, 37.5%)	diabetes type 1 (*n* = 1, 12.5%)	full recovery (*n* = 8, 100%)
*Marseglia* et al., 2006 [18]	*n* = 18	primary headache (*n* = 8, 44%) sinusitis (*n* = 8, 44%) CNS tumor (*n* = 1, 6%)	1–25 days	headache (*n* = 18, 100%)	none	none	none	none	fever (*n* = 7, 39%)	CT	S. aureus, S. pneumoniae	antibiotics (*n* = 18, 100%)	allergic rhinitis (*n* = 8, 44%) URTI (*n* = 4, 22%) swimming and diving (*n* = 3, 17%)	full recovery (*n* = 18, 100%)
*Inflammatory (acute/chronic)*														
*Caimmi* et al., *2011* [19]	*n* = 11	sinusitis (*n* = 8, 73%) tension-type headache (*n* = 3, 27%) CNS tumor (*n* = 3, 27%)	1–12 days	headache (*n* = 11, 100%)	CN III, V, VI, VII (*n* = 3, 27%)	nuchal rigidity (*n* = 5, 45%) stupor (*n* = 4, 36%)	none	none	fever (*n* = 9, 82%) nasal symptoms (*n* = 2, 18%)	CT	S. aureus, S. pneumoniae	antibiotics (*n* = 11, 100%) surgery (*n* = 3, 27%)	swimming and diving (*n* = 5, 45%) URTI (*n* = 5, 45%) alergic rhinitis (*n* = 3, 27%)	full recovery (*n* = 11, 100%)
*Kotowski and Szydlowski, 2023* [1]	*n* = 16	NR	6 weeks–30 months *	headache (*n* = 15, 94%)	NR	none	diplopia (*n* = 2, 12%) papilloedema (*n* = 1, 6%) visual acuity disturbances (*n* = 1, 6%)	none	nasal symptoms (*n* = 1, 6%)	CT + MRI	NR	surgery (*n* = 16, 100%)	NR	full recovery (*n* = 11, 69%) persistent headache (*n* = 5, 31%)
*Han* et al., *2024* [20]	*n* = 41	migraine headache (*n* = 24, 58.5%) unspecified headache (*n* = 11, 27%) tension-type headache (*n* = 2, 5%) secondary headache (*n* = 4, 10%)	≥3 months (*n* = 21, 51%) < 3 months (*n* = 20, 49%)	headache (*n* = 41, 100%)	NR	dizziness (*n* = 13, 32%) gait disturbance (*n* = 1, 2%)	photophobia, diplopia, blurred vision, ocular pain (*n* = 14, 34%)	none	nausea/vomiting (*n* = 23, 56%) respiratory symptoms (*n* = 9, 22%) otologic symptoms (*n* = 6, 15%) abdominal pain (*n* = 4, 10%)	CT + MRI	NR	analgesics/observation (*n* = 26, 68%) antibiotics (*n* = 12, 32%) surgery (*n* = 1, 4%)	allergy (*n* = 4, 10%)	full recovery (*n* = 26, 68%) persistent headache in untreated patients (*n* = 12, 32%) lost to follow up (*n* = 3)
*Strek* et al., *2007* [5]	*n* = 2	NR	NR	headache (*n* = 2, 100%)	CN III (*n* = 1, 50%)	meningeal signs (*n* = 1, 50%) dizziness (*n* = 1, 50%)	unspecified ocular symptoms (*n* = 1, 50%)	meningitis (*n* = 1, 50%)	nasal symptoms (*n* = 1, 50%)	CT	NR	surgery (*n* = 2, 100%)	NR	full recovery (*n* = 1, 50%) persistent bilateral oculomotor nerve palsy (*n* = 1, 50%)
*Inflammatory (acute/chronic)*														
*Strek* et al., *2007* [21]	*n* = 2	NR	NR	headache (*n* = 2, 100%)	CN III (*n* = 1, 50%)	meningeal signs (*n* = 2, 100%)	unspecified ocular symptoms (*n* = 1, 50%)	meningitis (*n* = 2, 100%)	fever (*n* = 2, 100%) nausea/vomiting (*n* = 2, 100%)	CT	NR	antibiotics (*n* = 2, 100%) surgery (*n* = 2, 100%)	NR	full recovery (*n* = 1, 50%) persistent bilateral oculomotor nerve palsy (*n* = 1, 50%)
*Guvenc* et al., *2009* [22]	*n* = 3	NR	2 days–3 weeks	headache (*n* = 2, 67%)	CN VI (*n* = 2, 67%)	orbital pain (*n* = 1, 33%)	diplopia (*n* = 2, 67%)	none	fever (*n* = 2, 67%)	CT + MRI	NR	antibiotics (*n* = 3, 100%) surgery (*n* = 2, 67%)	none	full recovery (*n* = 3, 100%)
*Sethi* et al., *1999* [23]	*n* = 2	NR	NR	headache (*n* = 2, 100%)	NR	NR	blurred vision (*n* = 1, 50%)	NR	NR	CT + MRI	NR	antibiotics (*n* = 2, 100%)	NR	full recovery (*n* = 2, 100%)
*Mucocele*														
*Uren* et al., *2003* [17]	*n* = 3	NR	10 days–more than 12 months	headache (*n* = 3, 100%)	none	none	reduced or blurred vision (*n* = 2, 67%) unilaterally reduced visual acuity(*n* = 2, 67%)	bone destruction (*n* = 1, 33%)	none	CT	S. aureus	surgery (*n* = 3, 100%)	facial injury (*n* = 1, 33%)	full recovery (*n* = 3, 100%)
*Kotowski and Szydlowski, 2023* [1]	*n* = 6	NR	6 weeks–30 months *	headache (*n* = 5, 83%)	none	none	visual acuity disturbances (*n* = 1, 17%)	none	none	CT + MRI	NR	surgery (*n* = 6, 100%)	NR	full recovery (*n* = 6, 100%)
*Strek* et al., *2007* [5]	*n* = 1	NR	NR	headache (*n* = 1, 100%)	none	none	none	none	nasal symptoms (*n* = 1, 100%)	CT	NR	surgery (*n* = 1, 100%)	NR	full recovery (*n* = 1, 100%)
*Tumors ect.*														
Kotowski and Szydlowski, 2023 [1]	*n* = 4	NR	6 weeks–30 months *	headache (*n* = 1, 25%)	NR	dizziness (*n* = 1, 25%)	none	NR	nasal symptoms (*n* = 1, 25%)	CT + MRI	lipoma (*n* = 1, 25%) osteoma (*n* = 1, 25%) chondrosarcoma (*n* = 1, 25%) sphenochoanal polyp (*n* = 1, 25%)	surgery (*n* = 4, 100%)	NR	full recovery (*n* = 1, 25%) persistent headache (*n* = 1, 25%) NR (2, 50%)
Sethi et al., 1999 [23]	*n* = 1	NR	NR	none	none	none	none	none	nasal symptoms (*n* = 1, 100%)	CT	polyp (*n* = 1, 100%)	surgery (*n* = 1, 100%)	NR	NR

**Legend:** * Kotowski and Szydłowski—the study provides data for the general population regardless of etiology. URTI—upper respiratory tract infection; CNS—central nervous system; CN—cranial nerve; CT—computer tomography; NR—not reported; MRI—magnetic resonance imaging; *n*—number of patients.

To reduce heterogeneity and provide a more clinically consistent profile of ISSD, data were stratified into three etiological subgroups: inflammatory lesions (121 patients, 89%), mucoceles (10 patients, 7.3%), and tumors (5 patients, 3.7%). In accordance with the eligibility criteria, studies reporting a single pediatric patient were excluded. However, larger case series and cohort studies were retained irrespective of the number of patients within specific pathological subgroups.

The following sections summarize the findings according to the predefined clinical domains of interest.

### 3.4. Reported Predisposing or Associated Factors

Data regarding predisposing or associated factors were reported in 7 out of the 11 included studies, encompassing a total of 99 patients. Individual patients could present with more than one risk factor. In the inflammatory group, reported factors included swimming/diving (11%), allergic rhinitis (14%), upper respiratory tract infection (10%), allergy (4%), prior sinusitis (1%), and type 1 diabetes mellitus (1%). In the mucocele group, facial injury was reported in one patient. Risk factors were not reported in the tumor group.

### 3.5. Initial Diagnosis

Data regarding initial diagnosis yielded 79 reported diagnoses among the 136 included patients. All reported initial diagnoses pertained to patients with inflammatory sphenoid sinus disease. The most common initial misdiagnosis was a primary headache disorder (*n* = 39, 49.4%), most frequently migraine. Other initial misdiagnoses included central nervous system tumors (*n* = 5, 6.3%) and central nervous system infections (*n* = 4, 5.1%). Sphenoid sinusitis was correctly identified in 16 out of the 79 reported initial diagnoses (20.3%).

### 3.6. Microbiological and Histopathological Findings

Detailed microbiological or histopathological data were available in 5 out of the 11 included studies. Bacterial pathogens were reported only in patients who underwent microbiological assessment. In the inflammatory and mucocele groups, identified pathogens included *Staphylococcus aureus* and *Streptococcus pneumoniae*. In the tumor group, histopathological evaluation revealed lipoma, osteoma, chondrosarcoma, and sphenochoanal polyp.

### 3.7. Time to Diagnosis

Time to diagnosis varied considerably across studies, ranging from 3 h to 30 months. In most studies, diagnosis was established within 1–6 weeks from symptom onset. Detailed results for patients with inflammatory etiologies are presented in Table 1.

Additionally, Uren et al. reported that among patients with mucoceles, time to diagnosis ranged from 10 days to more than one year, whereas Kotowski et al. and Szydłowski et al. did not specify diagnostic timing for individual pathological subgroups.

### 3.8. Presence of Headache

Headache was reported in 10 out of the 11 included studies and represented the most common clinical manifestation across all etiological groups. The prevalence of headache was 98% (119/121) in the inflammatory group, 90% (9/10) in the mucocele group, and 20% (1/5) in the tumor group. Overall, headache was reported in 129 out of 136 patients (94.9%).

### 3.9. Ocular Symptoms/Signs

Data regarding ocular manifestations were reported in 10 out of the 11 included studies. The spectrum of ocular symptoms included blurred vision, diplopia, photophobia, decreased visual acuity, and ocular pain. Ocular manifestations were observed in 30/121 patients (24.8%) in the inflammatory group and in 3/10 patients (30%) in the mucocele group, whereas no ocular symptoms were reported in the tumor group.

### 3.10. Other Neurological Symptoms

Data regarding additional neurological manifestations were documented in 7 out of the 11 included studies. Reported symptoms were heterogeneous, and individual patients frequently presented with multiple neurological manifestations.

In the inflammatory group, reported symptoms included nuchal rigidity (8.4%), periorbital pain (2.5%), stupor (5%), altered mental status (1.7%), dizziness (11.8%), gait disturbance (20.9%), and meningeal signs (2.5%). These findings were reported in 119 patients; data were missing for two patients. In the tumor group, non-focal neurological symptoms were reported in 20% (1/5) of patients.

### 3.11. Focal Neurological Symptoms

Detailed information regarding cranial nerve involvement was reported in 7 studies and involved 10 out of 54 patients with available data (19%). All affected patients belonged to the inflammatory group. Cranial nerves III, V, VI, and VII were involved most frequently.

### 3.12. Intracranial/Orbital Complications

Intracranial or orbital complications were identified in 4 out of the 11 included studies. Reported complications in the inflammatory group included meningitis and frontal lobe abscess, with an overall prevalence of 3.4% (4/119). In the mucocele group, complications occurred in 10% (1/10) of patients and included bone destruction involving the clinoid processes and pituitary floor.

### 3.13. Other Non-Neurological Symptoms

Non-neurological symptoms were reported in 10 studies and included fever, nasal symptoms (e.g., congestion or discharge), gastrointestinal symptoms (e.g., nausea, vomiting, abdominal pain), respiratory symptoms, and otologic symptoms.

The most common non-neurological symptom in the inflammatory subgroup was fever, reported in 36 patients (30.3%). Of note, sinonasal symptoms occurred in only 16/119 patients (13.4%). In the mucocele group, nasal discharge was reported in one patient (10%). In the tumor group, nasal symptoms were reported in two patients (40%), both of whom presented with polyps.

### 3.14. CT/MRI Imaging

In accordance with the eligibility criteria, all included patients had radiologically confirmed isolated sphenoid sinus disease. In 7 out of the 11 included studies, diagnosis was based exclusively on CT imaging. In the remaining studies, diagnosis was established using CT, MRI, or both modalities.

### 3.15. Treatment Strategy

Treatment strategies were reported in all included studies. In the inflammatory group, antibiotic therapy was administered to 73/121 patients (60.3%), representing the most common treatment modality. Preoperative antibiotic therapy was excluded from analysis, as it was not considered definitive treatment of the underlying pathology. Clinical resolution without surgery was reported in 27 patients (22.3%), including patients managed conservatively or symptomatically. Overall, surgical intervention was ultimately required in 31 patients (25.6%), including those initially treated with antibiotics.

All patients in the mucocele and tumor groups underwent surgical treatment. Functional endoscopic sinus surgery (FESS) was the most commonly performed procedure across all groups.

### 3.16. Long-Term Sequelae

Outcome data were available in all included studies, although follow-up duration and reporting remained heterogeneous. In the inflammatory group, full recovery was reported in 84% (99/118) of patients. Persistent sequelae included headache and bilateral oculomotor nerve palsy; three patients were lost to follow-up.

In the mucocele group, full recovery was reported in all patients, with no persistent neurological deficits, although interpretation is limited by the small sample size. In the tumor group, outcomes were heterogeneous, with persistent headache reported in some patients and incomplete follow-up data in three cases.

## 4. Discussion

In this systematic review, encompassing 136 pediatric cases from 11 studies, we demonstrate that isolated sphenoid sinus disease (ISSD) in children is frequently characterized by prominent neurological rather than sinonasal manifestations. Headache was the most frequent neurological symptom, occurring in 94.9% of patients regardless of underlying etiology. This observation is consistent with previous reports identifying headache as the leading clinical feature of ISSD, with rates of 98.6% reported by Clement et al. and 78% by Kotowski et al. [1,9]. These findings suggest that ISSD in children often follows a neurologically driven clinical presentation pattern, which may challenge traditional diagnostic approaches based primarily on sinonasal symptomatology [24].

Importantly, our analysis demonstrated that ocular manifestations were more frequent than otolaryngological symptoms (24.2% vs. 18.7%). Similar trends have been observed in previous studies [6,25]. For example, in the major pediatric case series by Kotowski et al., ocular symptoms were also more common than sinonasal manifestations (19% vs. 7.7%) [1].

A predominance of headache has likewise been consistently reported in adult populations with ISSD, where headache is recognized as the most common presenting symptom, often occurring in the absence of prominent sinonasal complaints. However, compared with pediatric cohorts, adult patients more frequently report concomitant nasal obstruction or discharge, resulting in a less pronounced predominance of neurological over sinonasal manifestations. Cranial nerve involvement and visual disturbances are also well-documented in adults but are usually associated with advanced disease or complications. Collectively, these findings may suggest a more pronounced neurological phenotype in pediatric ISSD [6,25,26,27].

A key finding of this review was the frequent initial consideration of diagnoses other than sphenoid sinusitis. Among the reported initial diagnoses, sphenoid sinusitis accounted for only approximately 20%, while primary headache disorders were the most common alternative diagnoses. The predominance of neurological and neuro-ophthalmological symptoms, together with the absence of typical EPOS-defined sinonasal features, likely contributes to diagnostic bias toward primary neurological, neuro-ophthalmological, or headache disorders [11,25,28]. According to the International Classification of Headache Disorders [8] (ICHD-3), headache attributed to disorders of the nose or paranasal sinuses represents a recognized category of secondary headache. Nevertheless, ISSD remains an underrecognized etiology of secondary headache in children, especially when sinonasal symptoms are absent or minimal.

The developmental anatomy of the sphenoid sinus may also contribute to diagnostic difficulty in children. Pneumatization progresses gradually during childhood, and the sphenoid sinus may therefore be less readily considered as a potential site of disease in younger patients.

Microbiological data were available in only a small proportion of patients, and inflammatory lesions should not be considered equivalent to microbiologically confirmed bacterial infection. When surgical drainage is performed, material obtained during the procedure can be used for microbiological assessment. Empirical antibiotic treatment was commonly used in inflammatory ISSD when microbiological confirmation was not available; however, the available data do not allow recommendations regarding a specific antibiotic regimen or treatment duration.

As emphasized in previous analyses, a high index of clinical suspicion is required to ensure timely diagnosis and prevent serious complications [25], including orbital cellulitis and abscess formation, persistent cranial nerve deficits, cavernous sinus thrombosis, and meningitis [6]. The limitations of conventional radiography further contribute to delayed diagnosis, as standard sinus X-rays do not adequately visualize the sphenoid sinus [29]. Consequently, ISSD is frequently diagnosed only after cross-sectional imaging (CT or MRI) is performed during the evaluation of persistent or atypical neurological symptoms. The main diagnostic pitfalls in pediatric ISSD include the predominance of headache, frequent ocular or neurological symptoms, and the absence or low frequency of sinonasal symptoms. This may lead to an initial diagnosis of migraine or another primary neurological disorder. In children with persistent or atypical headache, particularly when associated with visual symptoms, diplopia, cranial nerve deficits, fever, or abnormal neurological findings, sphenoid sinus disease should be considered. In such cases, early cross-sectional imaging with CT and/or MRI may help reduce diagnostic delay. CT provides rapid assessment of the sphenoid sinus and bony anatomy, whereas MRI is preferred when intracranial or orbital extension, cranial nerve involvement, or neoplastic pathology is suspected. Once sphenoid sinus disease is identified, early otolaryngological assessment is recommended, with ophthalmological or neurological evaluation depending on the clinical presentation [11,30].

Despite the growing literature on isolated sphenoid sinus disease, most available studies include predominantly adult populations or focus mainly on surgical and otolaryngological aspects. Pediatric data addressing neurological presentation, neuro-ophthalmological symptoms, secondary headache, and diagnostic pitfalls remain limited. To our knowledge, this study represents the first systematic review specifically focused on the neurological presentation and secondary headache profile of pediatric ISSD.

From a clinical perspective, our findings highlight the importance of considering ISSD in the differential diagnosis of neurological and neuro-ophthalmological symptoms in children. Persistent or atypical headache accompanied by ocular symptoms, cranial nerve deficits, fever, or abnormal neurological examination findings should prompt early consideration of sphenoid sinus pathology and appropriate neuroimaging, even in the absence of sinonasal complaints [25].

This study has several limitations. The literature search was limited to PubMed and Web of Science, which may have resulted in the omission of relevant studies, particularly given the rarity of pediatric ISSD, and should be considered when interpreting the completeness of the available evidence. The review protocol was not prospectively registered, which represents a methodological limitation and may increase the risk of selective reporting or post hoc changes, although predefined eligibility criteria, independent study selection, standardized data extraction, and a reproducible search strategy were used. Most included studies were small retrospective case series, and some included mixed adult and pediatric populations with limited availability of pediatric-specific data. Follow-up duration and outcome reporting were variable, which may have led to the underestimation of persistent neurological sequelae. Despite stratification by disease etiology, differences in patient selection, diagnostic criteria, symptom and outcome reporting, and follow-up remained substantial and limited direct comparison between studies. Exclusion of single-patient case reports may also have resulted in underrepresentation of the rarest neurological complications. Overall, these limitations reduce the certainty of the available evidence and should be considered when interpreting the findings.

## 5. Conclusions

Neurological manifestations frequently constitute the initial or predominant presentation of isolated sphenoid sinus disease (ISSD) in children and are associated with a risk of delayed diagnosis and significant morbidity. Although sinonasal symptoms may be present, neurological and neuro-ophthalmological features often dominate the clinical picture and may obscure the underlying etiology, contributing to diagnostic delay. In many cases, ISSD is identified on cross-sectional imaging performed during the evaluation of persistent or atypical neurological symptoms. Particular attention should be paid to ocular manifestations accompanied by headache, as they may indicate cranial nerve involvement and should be considered a clinical red flag warranting prompt neuroimaging and otolaryngological evaluation. Increased awareness of ISSD among pediatricians and neurologists may facilitate earlier diagnosis and reduce the risk of potentially irreversible neurological complications.

## Data Availability

No new data were created in this study. Data supporting the findings of this systematic review are available in the included published articles and in the Supplementary Materials.

## Supplementary Materials

The following supporting information can be downloaded at: https://www.mdpi.com/article/10.3390/brainsci16080897/s1, Table S1a [1,15,16,17,18,19,20]: Characteristics of included studies: pediatric population; Table S1b [5,21,22,23]: Characteristics of pediatric cases extracted from mixed-population studies; Table S2 [1,5,15,16,17,18,19,20,21,22,23]: Quality assessment of the included studies using the Joanna Briggs Institute (JBI) critical appraisal tools; Supplementary Data S1: Databases and search terms used in literature search.
