# Supplementary material for: Neurological Manifestations and Diagnostic Delay in Pediatric Isolated Sphenoid Sinus Disease: A Systematic Review
Source: Brain Sci. 2026 Aug 21;16(8):897. doi: 10.3390/brainsci16080897 (PMC13511559; doi:10.3390/brainsci16080897)
Supplement: Supplementary file 1 [file brainsci-16-00897-s001.zip › Supplementary Data S1. Databases and search terms used in literature search..pdf]

Supplementary Data S1. Databases and search terms used in literature search

Database	Search query
Web of Science<br>(1962 to January 3, 2026)	TS ("Sphenoid Sinus" OR "Sphenoid Sinusitis" OR sphenoiditis OR "isolated sphenoid sinusitis" OR "isolated sphenoid sinus disease")<br>AND<br>TS ("Cranial Nerve Diseases" OR "Neurologic Manifestations" OR "cranial nerve*" OR "cranial neuropathy" OR neurologic* OR ophthalmoplegia OR diplopia OR "vision loss" OR "visual impairment" OR meningitis OR abscess OR empyema OR "cavernous sinus thrombosis" OR intracranial)<br>AND<br>TS (Child* OR Infant* OR Adolescent* OR pediatric* OR paediatric* OR infant* OR adolescen*)
Pubmed<br>(1966 to January 3, 2026)	("Sphenoid Sinus"[Mesh] OR "Sphenoid Sinusitis"[Mesh] OR "sphenoid sinus"[Title/Abstract] OR "sphenoid sinusitis"[Title/Abstract] OR sphenoiditis[Title/Abstract] OR "isolated sphenoid sinusitis"[Title/Abstract] OR "isolated sphenoid sinus disease"[Title/Abstract]) AND("Cranial Nerve Diseases"[Mesh] OR "Neurologic Manifestations"[Mesh] OR "cranial nerve*"[Title/Abstract] OR "cranial neuropathy"[Title/Abstract] OR neurologic*[Title/Abstract] OR ophthalmoplegia[Title/Abstract] OR diplopia[Title/Abstract] OR "vision loss"[Title/Abstract] OR "visual impairment"[Title/Abstract] OR meningitis[Title/Abstract] OR abscess[Title/Abstract] OR empyema[Title/Abstract] OR "cavernous sinus thrombosis"[Title/Abstract] OR intracranial[Title/Abstract]) AND ("Child"[Mesh] OR "Infant"[Mesh] OR "Adolescent"[Mesh] OR child*[Title/Abstract] OR pediatric*[Title/Abstract] OR paediatric*[Title/Abstract] OR infant*[Title/Abstract] OR adolescen*[Title/Abstract])
