# Supplementary material for: Neurological Manifestations and Diagnostic Delay in Pediatric Isolated Sphenoid Sinus Disease: A Systematic Review
Source: Brain Sci. 2026 Aug 21;16(8):897. doi: 10.3390/brainsci16080897 (PMC13511559; doi:10.3390/brainsci16080897)
Supplement: Supplementary file 1 [file brainsci-16-00897-s001.zip › Table S1a.pdf]

Table S1a. Characteristics of included studies: pediatric population.

Autor	Year	Country	Total number of patients	Number of patients with ISSD	Age range years	M:F
Haimi-Cohen et al. [16]	1999	Israel	8	8	9-17	Male:4 (50%), Female:4 (50%)
Lahat et al. [15]	1997	Israel	10	10	8-14	Male:6 (60%), Female:4 (40%)
Uren et al. [17]	2003	Australia	11	11	7-16	Male:9 (82%), Female:2 (18%)
Marseglia et al. [18]	2006	Italy	18	18	7-15	Male:8 (44%), Female:10 (56%)
Caimmi et al. [19]	2011	Italy	27	11	8-17	NR
Kotowski et al. [1]	2023	Poland	28	26	5-17	Male:11 (42%), Female:15 (58%)
Han et al. [20]	2024	Korea	41	41	5-17	Male:24 (59%), Female:17 (41%)
