# Supplementary material for: Neurological Manifestations and Diagnostic Delay in Pediatric Isolated Sphenoid Sinus Disease: A Systematic Review
Source: Brain Sci. 2026 Aug 21;16(8):897. doi: 10.3390/brainsci16080897 (PMC13511559; doi:10.3390/brainsci16080897)
Supplement: Supplementary file 1 [file brainsci-16-00897-s001.zip › Table S1b.pdf]

Table S1b. Characteristics of pediatric cases extracted from mixed-population studies.						
Author	Year	Country	Total number of pts.	Number of pediatric pts.	Age range years	M:F
Sethi et al. [23]	1999	Singapore	21	3	15-18	Male:2 (67%), Female:1 (33%)
Strek et al. [21]	2007	Poland	22	3	13-18	Male:2 (67%), Female:1 (33%)
Strąk et al. [5]	2007	Poland	7	2	13-14	Male:1 (50%), Female:1 (50%)
Güvenc et al. [22]	2009	Turkey	10	3	9-13	Male:0 (0%), Female:3 (100%)
