# Supplementary material for: Neurological Manifestations and Diagnostic Delay in Pediatric Isolated Sphenoid Sinus Disease: A Systematic Review
Source: Brain Sci. 2026 Aug 21;16(8):897. doi: 10.3390/brainsci16080897 (PMC13511559; doi:10.3390/brainsci16080897)
Supplement: Supplementary file 1 [file brainsci-16-00897-s001.zip › Table S2.pdf]

Table S2. Quality assessment of the included studies using the Joanna Briggs Institute (JBI) critical appraisal tools.

Author	Q1	Q2	Q3	Q4	Q5	Q6	Q7	Q8	Q9	Q10	Risk of bias
Lahat, 1997 [15]	yes	yes	yes	yes	yes	yes	yes	yes	yes	yes	low
Haimi-Cohen, 1998 [16]	yes	yes	yes	yes	yes	yes	yes	yes	yes	yes	low
Sethi, 1999 [23]	unclear	yes	yes	unclear	unclear	yes	yes	unclear	yes	yes	moderate
Uren, 2003 [17]	yes	yes	yes	yes	yes	yes	yes	yes	yes	yes	low
Marseglia, 2006 [18]	yes	yes	yes	yes	yes	yes	yes	yes	yes	yes	low
Strąk, 2007 [5]	yes	yes	yes	unclear	unclear	yes	yes	yes	yes	yes	low
Strek, 2007 [21]	yes	yes	yes	unclear	unclear	yes	yes	yes	yes	yes	low
Güvenc, 2009 [22]	unclear	yes	yes	no	no	yes	yes	yes	yes	yes	moderate
Caimmi, 2011 [19]	yes	yes	yes	yes	yes	unclear	yes	yes	yes	yes	low
Kotowski, 2023 [1]	yes	yes	yes	yes	yes	yes	yes	yes	yes	yes	low
Han, 2024 [20]	yes	yes	yes	yes	yes	yes	unclear	-	-	-	low
